# Parasitism and the expression of sexual dimorphism

**DOI:** 10.1002/ece3.1416

**Published:** 2015-01-30

**Authors:** Stephen P De Lisle, Locke Rowe

**Affiliations:** Department of Ecology and Evolutionary Biology, University of TorontoToronto, Ontario, Canada

**Keywords:** *Clinostomum*, condition dependence, Hamilton-Zuk hypothesis, *Notophthalmus viridescens*, sexual dimorphism

## Abstract

Although a negative covariance between parasite load and sexually selected trait expression is a requirement of few sexual selection models, such a covariance may be a general result of life-history allocation trade-offs. If both allocation to sexually selected traits and to somatic maintenance (immunocompetence) are condition dependent, then in populations where individuals vary in condition, a positive covariance between trait expression and immunocompetence, and thus a negative covariance between trait and parasite load, is expected. We test the prediction that parasite load is generally related to the expression of sexual dimorphism across two breeding seasons in a wild salamander population and show that males have higher trematode parasite loads for their body size than females and that a key sexually selected trait covaries negatively with parasite load in males. We found evidence of a weaker negative relationship between the analogous female trait and parasite infection. These results underscore that parasite infection may covary with expression of sexually selected traits, both within and among species, regardless of the model of sexual selection, and also suggest that the evolution of condition dependence in males may affect the evolution of female trait expression.

## Introduction

Parasitism is ubiquitous in the wild and is often expected to play a key role in determining realized fitness. Thus, parasitism has been envisioned as playing a key role in a number of evolutionary problems, including the maintenance of female preference for male traits that do not appear to offer a direct fitness benefit to choosy females (Hamilton and Zuk 1982). An underappreciated point is that parasite load may covary with expression of any trait under directional sexual and net stabilizing selection, because these traits are expected to evolve reaction norms that couple trait expression with phenotypic quality (Nur and Hasson 1984; Rowe and Houle 1996; Bonduriansky 2007). Although individuals may face a trade-off between resource investment in sexually selected traits and investment in immunocompetence and other forms of somatic maintenance, among-individual variation in the size of the resource pool available for allocation allows some individuals to appear to conquer the trade-off (van Noordwijk and de Jong 1986) and thus can create positive phenotypic correlations between traits that are expec-ted to trade off. That is, regardless of the mechanism of sexual selection and even if individuals face a trade-off between allocation to immunocompetence and a sexually selected trait, a negative phenotypic covariance between parasite load and trait expression across males could be expected under the assumption that somatic maintenance for immunocompetence is itself condition dependent. Thus, even for traits where mating biases are determined by competition among males for mating opportunities, rather than actual female preference, a relationship between parasite load and trait expression may be expected if condition-dependent expression of the trait is favored; higher condition individuals may have both higher immunocompetence, thus lower parasite load and greater expression of the sexually selected trait.

Further, sexual dimorphism in parasite infection is expected to be commonplace, because parasite infection is an outcome of optimal investment in somatic maintenance and time allocated to life stages vulnerable to infection, both of which may often differ between males and females because the sexes themselves are defined by their divergent life-history strategies of gamete investment (Shärer et al. 2012). There is, in fact, a substantial literature on sexual dimorphism in parasite loads and immunocompetence. Most of the empirical work indicates that males usually have higher parasite loads or lower immunocompetence than females (Poulin 1996). Several proximate explanations have been constructed to explain this empirical pattern (Folstad and Karter 1992; Rolff 2002). Yet optimal life-history models indicate that although sexual selection typically does lead to a decrease in optimal male investment in immunocompetence relative to females, higher female immunocompetence is not an inevitable evolutionary outcome (Stoehr and Kokko 2006). Thus, the generality of male bias in parasite loads in the wild is still an open question.

**Figure 1:**
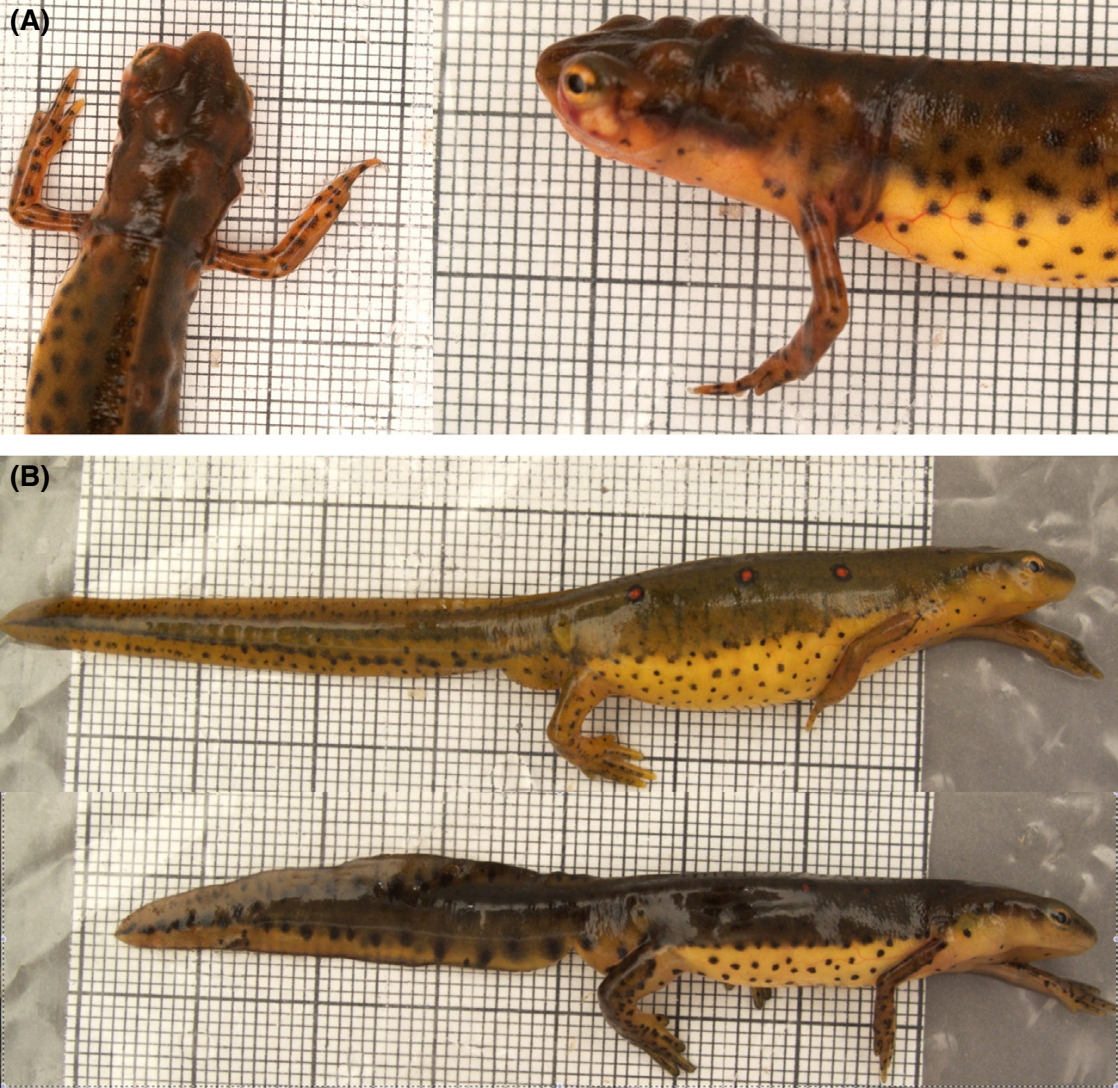
(A) *Clinostomum* infection in red-spotted newts, *Notophthalmus viridescens*. Newts are susceptible to infection by *Clinostomum* larvae, which encyst to form metacercariae, visible on this heavily infected individual as distinct bumps. (B) An illustration of sexual dimorphism in tail height in newts (top: female; bottom: male). Males with taller tails have greater success in premating struggles.

**Figure 2:**
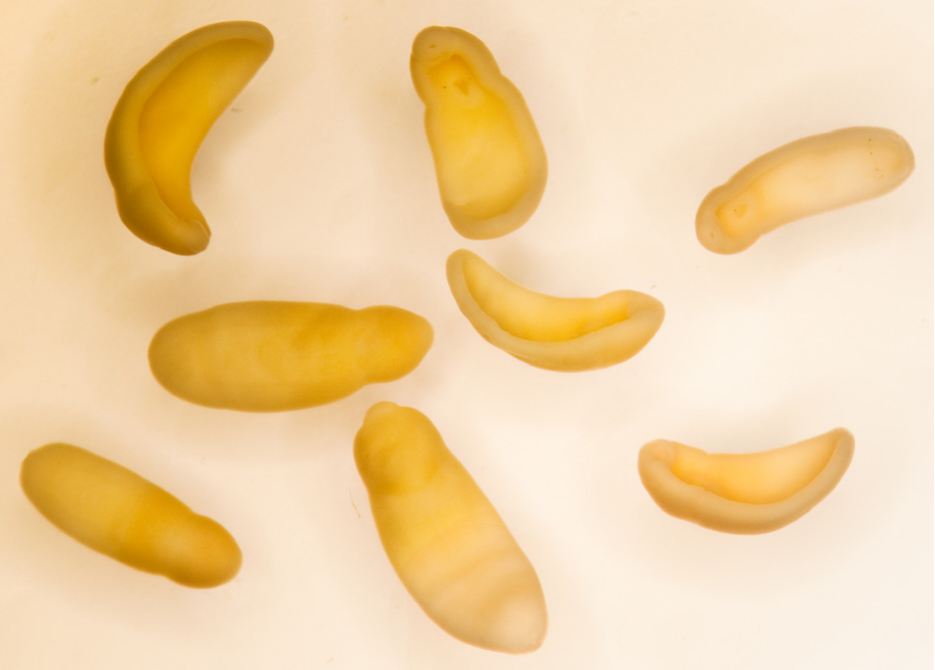
A sample of *Clinostomum* metacercariae that emerged from a heavily infected adult male newt upon anesthetization. The mass emergence was fatally traumatic for the newt. Metacercariae were preserved in 95% ethanol and later photographed at 10× magnification.

In this study, we survey a wild population of red-spotted newts, *Notophthalmus viridescens*, and examine whether expression of a key trait under sexual selection via scramble competition among males (Able 1999) covaries with parasite infection, as predicted by the hypothesis that expression of condition-dependent sexually selected traits should covary with parasite load regardless of the model of sexual selection. Because mating bias toward tall-tailed males is not determined by female preference in newts (Gabor et al. 2000), classic models of parasite-mediated coevolution would make no speci-fic prediction for such a relationship. Further, unlike many other studies focused on male-limited display traits (Møller et al. 1999), we were also able to estimate the relationship between female parasite load and variation in female expression of the sexually selected (in males) trait at its naturally selected optimum. Wild populations of newts can harbor a diversity of parasites, but here we focus on trematode worms of genus *Clinostomum*, whose larval stage can encyst in adult newts, forming plainly visible metacercariae cysts (Figs.1A, 2).

## Methods

### Study system

Red-spotted newts are a common pond-breeding salamander characterized by their complex mating system, where males pursue reluctant females and clasp them in premating struggles aimed at increasing female receptivity (Verrell 1982). Male tail height (Fig.1B) is a key trait that aids in these struggles, and males with taller tails have higher mating success in both *Notophthalmus* (Able 1999; Gabor et al. 2000; Bloch and Grayson 2010) and the sister clade (Janzen and Brodie 1989).

Newts in the wild are infected by a number of parasites (Raffel et al. 2008), particularly flatworms of class Trematoda (Muzzall 1991), including the genus *Clinostomum* (in North American, *C. marginatum*). *Clinostomum* have a complex life cycle in which eggs are ingested by snails, which then hatch and emerge from the snail as larvae that then encyst below the epidermis of a fish or amphibian intermediate host (Hopkins 1933; Klass 1963). If this intermediate host is consumed by an endothermic definitive host, typically a bird (Klass 1963; but see Chung et al. 1995), the *Clinostomum* metacercariae emerge as adults and reproduce in the digestive tract of the host. *Clinostomum* infections are likely costly for newts; heavy infections concentrated around the head and mouth (Fig.1A) may disrupt feeding and use a substantial component of the hosts resource pool. Further, because newts are toxic and generally avoided by endothermic predators, it is possible that parasite emergence occurs before host death. Such an emergence was observed in our study in two instances which where both fatally traumatic for the host, and scars on wild-caught individuals (S. De Lisle, personal observation) suggest such premature parasite emergence may occur in the wild.

### Survey

Aquatic adult newts (101 males and 112 females) were captured from a single pond at the Koffler Scientific Reserve (King, Ontario) between 14 June and 20 June 2012, at the approximate peak of their local breeding season, and also on 22 May and 28 May 2014 (104 males and 96 females). We followed the same measurement procedures in both years, and individuals with low or zero infection were used in other unrelated experiments (De Lisle and Rowe 2014) following measurement. Newts were anesthetized for measurement of body mass, body length, and tail height (at the tallest point on the tail), and parasite load. Mass was measured by first gently patting each newt with a paper towel to remove excess moisture; linear measurements were taken to two decimal places with digital calipers by the same technician (SD). Trematode parasite load was estimated by counting the number of visible *Clinostomum* metacercariae (Fig.1A). Although some metacercariae were probably missed using this approach as opposed to dissection, especially for heavily infected individuals, we were able to obtain an estimate of parasite load that avoided euthanasia. As a caveat, it is certainly probable that other parasite taxa infect newts in the study population. However, we observed no other macroparasites, and the high morbidity and prevalence of *Clinostomum* infections suggest they are an important parasite in this population.

### Statistical analysis

We used a generalized linear model (GLM) to assess the effect of sex, body length, and their interaction on the number of *Clinostomum* metacercariae. Including body length as a covariate allowed us to assess any effect of sex on parasite abundance independent of any differences due to sexual size dimorphism. We dropped the sex*body length interaction when not significant to test main effects, as is appropriate in ANCOVA-type linear models to test significance of categorical main effects (Engqvist 2005). We were also interested in assessing the relationship between infection load and tail height, a sexually selected trait in newts. Because parasite load and tail size both covary with body size, we first obtained a size-corrected estimate of individual tail height by rotating the data in a principle component analysis of body length and tail height, where PC 2 represents tail height variation independent of body size (PC 1). Analyzing this rigid rotation of the data is biologically appropriate because size-corrected tail height is under directional sexual selection (Able 1999). Further, this approach avoids collinearity issues that could be associated with including both body size and tail height in the same multiple regression. We then performed a GLM to estimate the effect of size-corrected tail height on parasite load; we performed separate analyses for males and females (including the PCA) and excluded one female from this analysis in 2014 whose tail height was over 5 standard deviations from the mean. We fit all GLMs separately by year, and the residual error was treated as negative binomial, accommodating over dispersion. Although it would be possible to include all years into a single generalized mixed model with random intercepts, slopes, and residual variance among years, we avoided this complicated random effect structure and so also avoid the subsequent issue of whether separate or pooled PCAs should be performed. Our approach of performing separate analyses for each year is conservative and does not represent an issue of multiple testing because the same biological hypothesis (but different statistical hypotheses) is being tested in each year. Analyses were performed in SAS v. 9.3 (Sas Institute Inc., Cary, NC); GLMs were fit in proc GLIMMIX.

## Results

**Figure 3:**
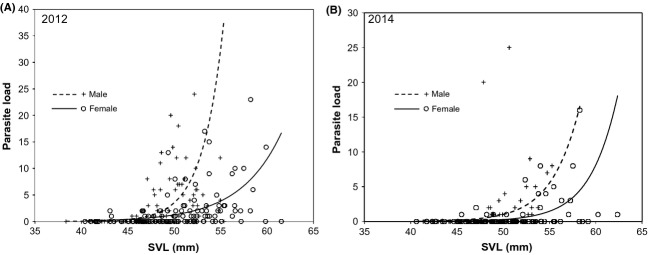
Parasite load, the number of visible *Clinostomum* metecercariae, of individuals in a wild population of red-spotted newts sampled across two years. Parasite load increased with body length in both years (A, 2012: sex*svl interaction *F*_1,209_ = 5.77, *P* = 0.0172; svl effect *F*_1,209_ = 53.44 *P* <0.0001; B, 2014: svl effect *F*_1,197_ = 13.83 *P* = 0.0003; interaction NS) and overall males had higher loads than females in both years (A, 2012: sex effect *F*_1,209_ = 4.8 *P* = 0.029; B, 2014: *F*_1,197_ = 4.69 *P* = 0.031). From GLMs with negative binomial error.

**Figure 4:**
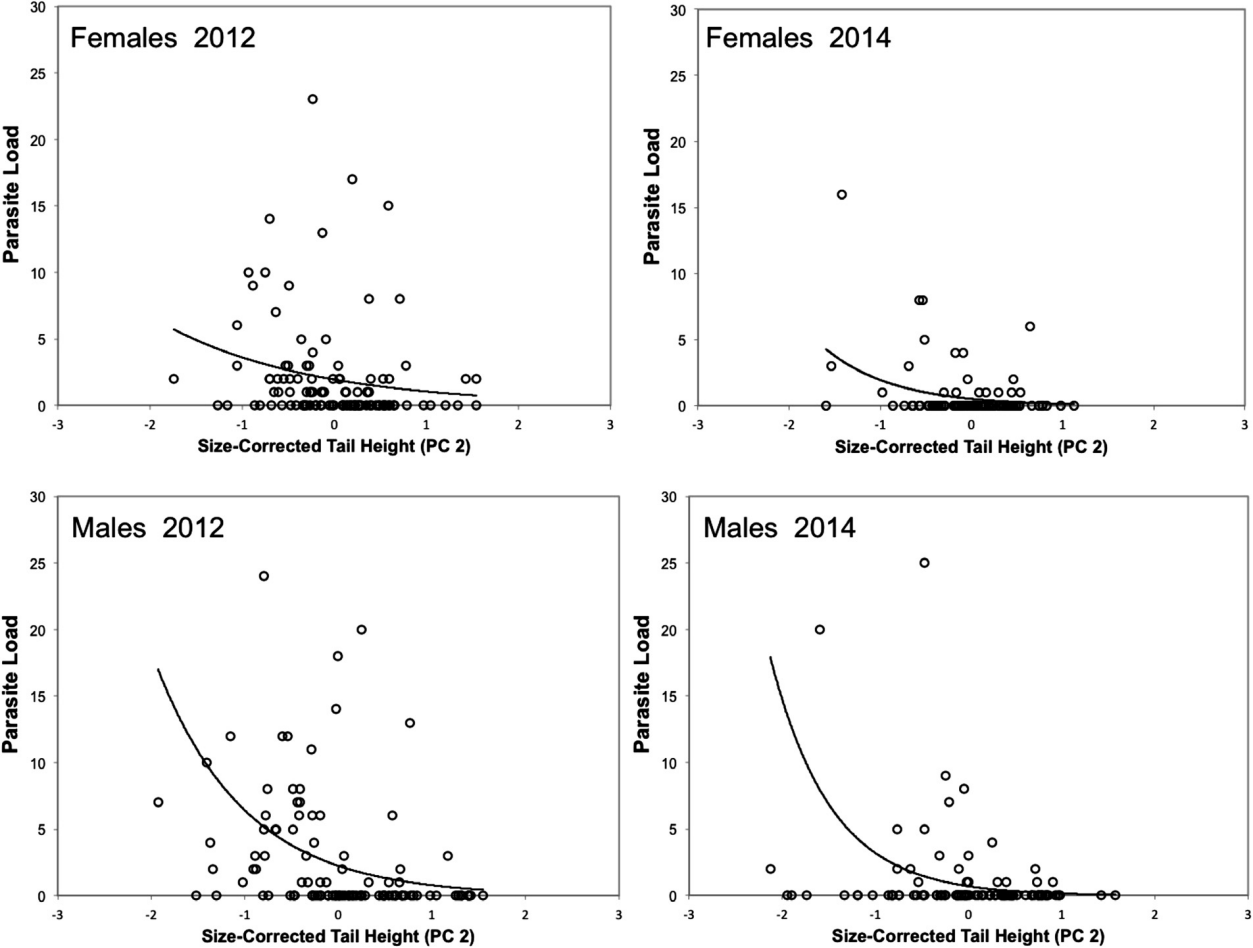
Relationship between size-corrected tail height (PC 2 of a PCA on tail height and body length), a sexually selected trait in males, and parasite load, the number of visible *Clinostomum* metacercariae. There was a significant negative relationship between tail size and parasite load in males in both years (2012: *F*_1,99 _= 9.91, *P *=* *0.0022; 2014: *F*_1,102 _= 9.16, *P *=* *0.0031). The analogous trait in females showed a weaker, but significant, negative relationship with parasite load in both years (2012: *F*_1,110 _= 3.67, *P *=* *0.0578; 2014: *F*_1,93 _= 6.14, *P *=* *0.015). From GLMs with negative binomial error.

Of the 213 newts captured and measured in 2012, 45 males and 55 females had at least one visible metacercariae; the proportion infected did not differ between the sexes (2 × 2 contingency table *χ*^2^_1_ = 0.44, *P *=* *0.51). Of the 200 newts captured in 2014, 22 males and 19 females had at least one visible metacercariae (2 × 2 contingency table *χ*^2^_1_ = 0.057, *P *=* *0.81). This among year difference in infection rates could be due to any number of factors, including temperature, seasonality, or chance. In two instances in 2012, both involving heavily infected male newts, *Clinostomum* metacercariae began a mass emergence following anesthetization of their host (Fig.2) that led to death of the newt hosts. Male newts had higher parasite loads than females in both years (2012: sex effect *F*_1,209_ = 4.8, *P *=* *0.029; 2014: *F*_1,197_ = 4.69, *P *=* *0.031; Fig.3) independent of body size (2012: sex*svl interaction *F*_1,209 _= 5.77, *P *=* *0.0172; svl effect *F*_1,209 _= 53.44, *P *<* *0.0001; 2014: svl effect *F*_1,197_ = 13.83, *P *=* *0.0003; interaction not significant, *F*_1,196_ = 0.08, *P *=* *0.78 and so dropped from model; Fig.3). Size-corrected tail height was negatively related to parasite load in males (2012: *F*_1,99 _= 9.91, *P *=* *0.0022; 2014: *F*_1,102 _= 9.16, *P *=* *0.0031) and females (2012: *F*_1,110 _= 3.67, *P *=* *0.0578; 2014: *F*_1,93 _= 6.14, *P *=* *0.015) in both years, although the relationship was consistently stronger in males (Fig.4).

## Discussion

We surveyed parasite infection of individuals in a wild salamander population across two years and demonstrate first that males have consistently higher trematode parasite loads than do females and second that parasite infection covaries negatively with tail height, a sexually selected trait in males. Our results support the hypothesis that parasite infection may generally covary with the expression of sexual dimorphism and that the sexes may often differ in total parasite load; our results are also consistent with a fairly large body of work suggesting males may often carry higher parasite loads than females.

Sexual dimorphism in total parasite load can arise due to sex differences in resource allocation to immunocompetence or as a result of sexual differences in encounter rates with parasites. Although these pathways are often treated in the literature as separate agents of causality (e.g., Stoehr and Kokko 2006), we view them as tightly linked because sex differences in parasite encounter rates can arise, for example, due to differences between the sexes in time spent in different life stages, itself a component of the life history. Although “immunocompetence” is itself a vague term, at some level it nonetheless represents an outcome of resource allocation to somatic maintenance. Thus, because parasite presence represents one cost determining the optimum time allocation to a life stage during ontogeny (e.g., Rowe and Ludwig 1991), parasite infection represents the total outcome of both time allocated to a life stage vulnerable to infection as well as resources allocated to immunocompetence and somatic maintenance, both of which may be shaped by selection in different ways for males and females. In newts, males of some populations are more likely to overwinter in the aquatic stage, where they are vulnerable to parasite infection, than are females that often migrate out of ponds and overwinter on land (Grayson and Wilbur 2009; Grayson et al. 2011). However, sex differences in migration propensity do not appear to be present in the population of this study (De Lisle and Rowe 2014). More importantly, parasites are probably rarely encountered in the winter because parasite loads reach their peak in the late spring/early summer (Raffel 2006). However, females in several populations appear more likely to skip years of reproduction (Gill 1978; Grayson et al. 2011) and thus exposure to the peak of parasite abundance, which may contribute to sex differences in parasite loads. Finally, male and female newts differ in habitat use within ponds; males appear to spend more time in limnetic habitat, while females are more frequently found in the benthos (Grayson et al. 2012). It is also possible that sex differences in immunocompetence play a role in the sex difference in parasite loads that we observed. Although our data do not allow these alternatives to be distinguished, sexual dimorphisms in parasite load in newts and many other taxa (e.g., Reimchen and Nosil 2001) probably reflect a complicated outcome of divergences in life history, ecology, and immune investment.

Relationships between trait expression and parasite load are often interpreted in light of models of parasite-mediated sexual selection, where female preference is maintained either via indirect genetic benefits sustained through host–parasite coevolution (Hamilton and Zuk 1982) or via direct selection to avoid parasites (Able 1996). In newts, mating advantage for males with tall tails appears to arise from scramble competition among males for access to mating opportunities (Verrell 1983; Able 1999) rather than female preference (Gabor et al. 2000), and the complex life cycle of *Clinostomum* precludes transmittance between parents and offspring. Thus, our results support the hypothesis that parasite infection may often covary with expression of sexually selected traits even if parasites play no major role in the maintenance of female preference.

We found evidence for a weak relationship between female parasite load and tail size, suggesting a similar but weaker pattern to that found in males. In many organisms, some sexually selected male traits are not completely sex-limited in expression, in that an analogous female trait may be expressed at or closer to the optimum favored by natural selection as is the case with newt tail height. Few studies examine the relationship between the expression of female analogs of sexually selected traits and female parasite loads. Such a relationship may suggest the presence of intralocus sexual conflict over the reaction norm of the trait, if the evolution of condition-dependent expression in males also leads to an increase in condition-dependent expression in females (Bonduriansky and Rowe 2005). Such a relationship could also suggest intralocus conflict over immune investment, if the optimal relationship between condition and immune investment differs between the sexes. Both genetic conflicts could affect the net fitness effect of mating bias for females (Gorton 2012; Long et al. 2012). In fact, sexual dimorphism in optimal immunocompetence generated by sexual selection (Stoehr and Kokko 2006) could generally lead to intralocus sexual conflict over optimal immune investment, eliminating any “good-genes” effects of female preference as envisioned by Hamilton and Zuk (1982) and others since (Møller and Alatalo 1999; Møller et al. 1999).

Our work illustrates that parasite infection may be generally related to sexual dimorphism within species. Further, hypotheses of parasite-mediated sexual selection predict a positive relationship between parasite load and sexual dimorphism among species (Hamilton and Zuk 1982). Yet this pattern too has a more parsimonious explanation, suggested originally by George Williams (Williams 1966a,b; Partridge and Endler 1987); sexually selected traits are part of the reproductive effort and should covary with life history and thus optimal investment in somatic maintenance (immunity), among species. That is, for species with low residual reproductive value, selection would favor increased allocation to current reproductive effort (including sexually selected traits in males) and reduced allocation to maintenance of the soma (including immune investment); the opposite would be true for taxa with high residual reproductive value. Thus, the most readily testable predictions of hypotheses of parasite-mediated sexual selection have immediate alternative explanations, which underscores Balenger and Zuk's (2014) emphasis that tests of parasite-mediated sexual selection should instead focus on the dynamics of host–parasite coevolution. Although parasites do represent a cost that may influence the evolution of life histories, including the expression of male reproductive effort, relationships between dimorphism and parasite load within and among species do not necessarily imply a direct role for host–parasite coevolution in mediating sexual selection. Concomitantly, this suggests a broader view of the role of parasitism in sexual selection and the evolution of sexual dimorphism that does not depend on a specific model of sexual selection.
